# Fractures in people with epilepsy: A nationwide population‐based cohort study

**DOI:** 10.1002/epi4.12776

**Published:** 2023-06-25

**Authors:** Marija Babunovska, Aleksandar Jovanovski, Bojan Boskovski, Marta Foteva, Igor Kuzmanovski, Gordana Kiteva Trencevska, Emilija Cvetkovska

**Affiliations:** ^1^ University Clinic for Neurology, Faculty of Medicine Ss. Cyril and Methodius University Skopje North Macedonia; ^2^ Clinic for Neurology, Early Rehabilitation and Complex Neurological Treatment Evangelical Clinics Gelsenkirchen Germany; ^3^ University Clinic for Orthopedic Surgery Ss. Cyril and Methodius University Skopje North Macedonia

**Keywords:** antiseizure medications, fractures, people with epilepsy, population‐based study

## Abstract

**Objective:**

The objective of this study was to determine the age, gender, and site‐specific prevalence of fractures in people with epilepsy (PWE) and matched general population comparators in a nationwide study in North Macedonia between 2015 and 2018.

**Methods:**

PWE and matched controls were selected through a systematic search of the electronic National Health System (eNHS). We determined the period prevalence (PP) of all site‐specific fractures. We also calculated gender and age‐specific incidence rate ratios (IRR) for various fractures. Odds ratios (ORs) and risk ratios (RR) were estimated for the number and type of ASM as well as comorbid conditions.

**Results:**

Out of 13 818 prevalent epilepsy cases, 6383 (46.2%) were females, and 7435 (53.8%) were males. 109 PWE out of 1000 had at least one fracture during the study period and ~8 people out of 1000 in the general population. The most frequent sites in terms of PP both in PWE and controls, were fractures of the lower arm, hip and femur, and lower leg. Significant differences in PP for all fracture locations were observed between PWE and controls (*P* < 0.001). The noticeable differences of ∼100 times higher PP were observed for fractures of the skull and jaw in PWE. IRR of any fracture in PWE was 272.84/10 000 person‐years; higher in the older age groups and among people who received >2 ASM. Fracture risk was increased with the use of >2 ASM (OR: 1.56; 95% CI: 1.32‐1.84 and RR: RR: 1.32). The presence of comorbidities also increased fractures risk (OR: 1.24; 95% CI: 1.10‐1.38).

**Significance:**

This population‐based study depicts a higher fracture prevalence in PWE compared to the general population. A higher number of ASM and the presence of comorbidities increase the risk of fractures and targeted prevention might be needed in those subgroups of PWE.

**Key Points**

- PWE have higher prevalence of bone fractures compared to the general population.
- The most frequent fracture sites for PWE are lower arm, hip and femur, and lower leg.
- The use of more than two ASM is associated with a higher risk of any fracture in PWE.
- The presence of comorbidities increases odds of fractures in PWE.

## 1 INTRODUCTION

Bone fractures can lead to disability and impaired quality of life, and in people with epilepsy (PWE) are associated with an elevated mortality rate.^1^, ^2^ Fractures in PWE might be result of seizure‐related impairment of consciousness followed by falls and fall‐related injuries.^3^, ^4^, ^5^ Vigorous muscle contractions during generalized tonic‐clonic seizures (GTCS) can cause joints dislocations and fractures as well.^3^ Furthermore, chronic antiseizure medication (ASM) treatment impedes bone metabolism, which increases bone fragility and elevates fragility fracture risk.^6^, ^7^, ^8^, ^9^, ^10^ A significant portion of PWE have various comorbidities, which may additionally increase the risk of falling and injury.^11^, ^12^, ^13^, ^14^ Therefore, PWE are more likely to suffer a serious fracture than the general population. However, despite possible debilitating consequences, little is known regarding the frequency, age and gender predominance, and locations of bone fractures in PWE. Small number of studies have examined the prevalence of fractures in PWE. Existent literature consists dominantly of case reports and case series or assessment of certain populations or age groups.^7^, ^15^, ^16^ The earlier data from large population‐based studies should be updated, especially since fracture risk may have been influenced by the introduction of newer ASM, availability of bisphosphonates for treatment of osteoporosis, and better treatment of other epilepsy comorbidities.^17^, ^18^, ^19^ More current information on fracture burden in PWE will provide essential information to guide patient counseling and develop effective preventions in the age groups with the highest occurrence. Therefore, we determined the age, gender, and site‐specific prevalence of fractures in PWE and matched general population comparators in a nationwide study in North Macedonia (N. Macedonia) between 2015 and 2018. Further, we estimated the risk of bone fractures in relation to the number and different types of ASM.

## 2 MATERIALS AND METHODS

### 2.1 Setting and data sources

We conducted a retrospective cohort study using the data from Electronic National Health System (eNHS). Macedonian residents have universal public health insurance under National Health Insurance Fund (NHIF), the single‐payer for medical services. The еNHS was established in 2013 and is hosted by the Ministry of Health. It contains demographic data, electronic health records (EHR) of inpatient and outpatient managed at all levels of healthcare facilities, drug prescriptions, procedural codes, and healthcare cost information.^20^, ^21^

### 2.2 Study population

A person with prevalent epilepsy was anyone with a diagnosis of epilepsy (ICD‐ 10 code G40.0‐9) and a prescription for any antiseizure medication (ASM). This definition is following the recommendations for epilepsy ascertainment in epidemiologic studies from the International League Against Epilepsy (ILAE) and was employed in the previous study by the same team.^22^, ^23^ We identified 13 825 PWE in N. Macedonia between January 1, 2015, to December 31, 2018 (the selection process is detailed in Figure S1). We collected demographic data and further divided them into three age groups: children and adolescents (0‐19 years), young adults (20‐49 years), and late midlife and elderly (older > 50 years). Seven individuals lacked information regarding their gender and age, as indicated in Table 1. Furthermore, an additional 128 patients were missing information solely regarding their permanent residence.

**TABLE 1:** Baseline characteristics of PWE and general population comparator group.

Characteristics of the study participants	PWE (*n* = 13 825)		Controls (*n* = 71 340)	
	*N*	%	*N*	%
Age (years), mean	41.9		42.2	
Age (years)				
<19	2899	21	13 275	18.6
20‐49	5164	37.4	28 878	40.5
>50	5755	41.6	29 187	40.9
Missing	7			
Gender				
Male	7435	53.8	37 522	52.6
Female	6383	46.2	33 818	47.4
Missing	7			
Number of antiseizure medications				
1 or 2 antiseizure medications	12 426	89.9	/	
3 or more antiseizure medications	1399	10.1	/	

### 2.3 Cases

During the study period, 1507 PWE had a total of 1735 bone fractures (ascertained by ICD codes S.00‐99). Fractures were classified by location into seven subgroups: scull; jaw; vertebrae; shoulder and upper arm; lower arm; hip and upper leg; and lower leg (Figure S1). To identify possible causes and the circumstances as the cause of fracture among PWE, we conducted a search using the following ICD‐10 codes: M80 osteoporosis with current pathological fracture; U50‐U72 sport activity of injured person at the time event occurred; U73 occupational activity of injured person at the time the event occurred; V00‐99 injuries sustained in transport accidents; a W00‐W49 falls and exposure to inanimate forces. We excluded patients with pathological fractures due to malignancy (M84.5).

PWE were further classified into two groups: those who were prescribed one or two ASM and those who were prescribed three or more ASMs throughout the study period. The number of ASMs is a criterion used by the ILAE to define drug‐resistant epilepsy (DRE).^24^ Additionally, a recent study has provided validation for several claims‐based definitions of DRE, including the prescription of more than two distinct ASMs.^25^ Although establishing a definitive diagnosis of DRE solely based on the available dataset is challenging, the presence of three or more ASMs may still indicate difficult‐to‐treat epilepsy in considerable number of patients. Additionally, we assessed the potential impact of enzyme‐inducing ASM (EIASM) and nonenzyme‐inducing ASM (non‐EIASM) on the likelihood of bone fractures.

### 2.4 Controls

For each PWE with comprehensive demographic data, we conducted a search within the еNHS population to identify individuals without epilepsy who matched in terms of age (±2 years), gender, and permanent residence. From this entire matching cohort, we randomly selected up to six individuals for each PWE and included those 71 340 control subjects in the final analysis.

**FIGURE 1:**
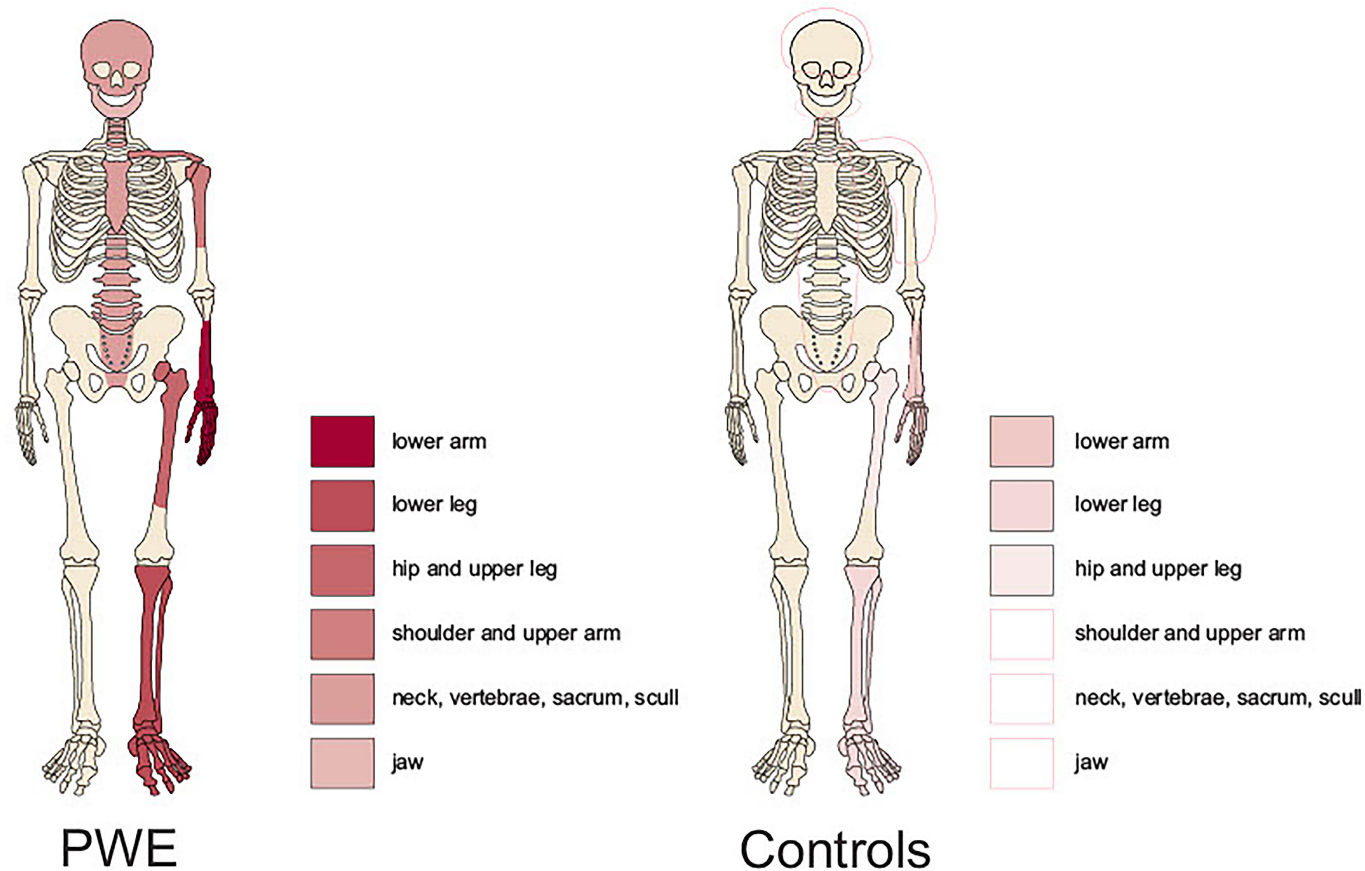
Schematic illustration the distribution and frequency of skeletal fractures (by the seven regions) in PWE and control subjects.

Based on the ICD codes (ICD‐10 codes S.00‐99), 565 controls had a total of 583 fractures during the study period (Figure 1).

### 2.5 Comorbidity

To evaluate the possible role of comorbidities in fracture risk among PWE and controls, we collected data on following conditions: stroke (I60‐I69), brain tumor (C70‐C72, D32‐D33), dementia (F00‐F03; G30‐ G32), osteoporosis (M80‐M82), intellectual disability (F70‐F79), cerebral palsy (G80‐G83), alcohol and substance abuse (F10‐F20), movement disorders (G20‐G21), multiple sclerosis (G35) and depression (F32‐F33).

### 2.6 Statistical analysis

The period prevalence (PP) of fractures (presented as a total number and divided by anatomic location and age group) was calculated among the selected PWE and control patients for the defined study period. In addition, a stratified analysis by gender was also conducted. Adjusted (for age and sex) and unadjusted ORs for fracture outcome for the number of ASM were calculated using logistic regression. The calculated odds ratios reflect odds of having a fracture for the unexposed (PWE using two or less ASM) compared to the exposed (PWE using three or more ASM), while considering the PWE with two or less ASM as reference. For the purpose of in‐depth analysis, using the already defined groups of exposed and unexposed, we calculated both risk and risk ratio applying a simple 2 by 2 table. We also calculated the ORs for fracture outcomes comparing EIASM to non‐EIASM, and separately for each of the most commonly utilized ASM. ORs of fractures were calculated in relation to the combination of epilepsy and different comorbidities.

Furthermore, we calculated incidence rates of different fracture types as the number of fractures divided per person‐time among PWE for the period of 2015‐2018. These analyses were stratified considering age group, gender and number of ASM. All analyses were conducted using STATA statistical software (version 13, StataCorp LP).

### 2.7 Ethics

The study was approved by the Ethics Committee of the University Clinic for Neurology. The present data cannot be shared, for legal reasons. Applications to access the Macedonian e‐health data must be submitted to the Department for E‐Health at the Ministry of Health (www.mojtermin.mk).

## 3 RESULTS

Characteristics of cases and controls are summarized in Table 1. Among the 13 825 PWE, 53.8% were male, and due to matching, a similar male predominance was seen among controls. The mean age at cohort entry was 41.9 years for the PWE and 42.2 years for controls. The distribution of cases and controls in each age group was similar.

### 3.1 Period prevalence of fractures in PWE and control subjects

Table 2 shows the total and separate age‐groups period prevalence (PP) for site‐specific fractures in PWE and control subjects. The most frequent sites in terms of PP both in PWE and controls, were fractures of the lower arm, hip and femur, and lower leg. Significant differences in PP for all fracture locations were observed between PWE and control subjects (*P* < 0.001). Nevertheless, the most remarkable difference between PWE and controls was observed for fractures of the skull and jaw. Schematic illustration of the distribution and frequency of skeletal fractures (by the seven regions) in PWE and control subjects are shown in Figure 1.

**TABLE 2:** The overall and separate age‐groups period prevalence (PP) for site‐specific fractures in PWE and control subjects.

People with epilepsy (PWE), (*n* = 13 825)				Controls, (*n* = 71 340)		
Types of fractures	Number of fractures	%	Period prevalence per 1000 inhabitants	Number of fractures	%	Period prevalence per 1000 inhabitants
People with at least one fracture	1507		109.01	565		7.92
Age > 19	248	16.5	85.55	133	23.5	10.02
Age 20‐49	473	31.4	91.60	145	25.7	5.02
Age > 50	786	52.2	136.58	287	50.8	9.83
Fractures of the scull	195		14.10	9		0.13
Age > 19	26	13.3	8.97	2	22.2	0.15
Age 20‐49	95	48.7	18.40	5	55.6	0.17
Age > 50	74	38	12.86	2	22.2	0.07
Fractures of the jaw	54		3.90	3		0.04
Age > 19	7	13	2.41	/	/	/
Age 20‐49	29	53.7	5.61	2	66.7	0.07
Age > 50	18	33.3	3.13	1	33.3	0.03
Fractures of the neck, vertebrae and sacrum	195		14.10	56		0.78
Age > 19	6	3.1	2.07	4	7.1	0.30
Age 20‐49	67	34.4	12.98	23	41.1	0.80
Age > 50	122	62.5	21.20	29	51.8	0.99
Fractures of the shoulder and upper arm	219		15.84	80		1.12
Age > 19	36	16.4	12.42	26	32.5	1.96
Age 20‐49	61	27.9	11.81	10	12.5	0.35
Age > 50	122	55.7	21.20	44	55	1.51
Fractures of the lower arm	501		36.24	193		2.71
Age > 19	134	26.7	46.22	75	38.9	5.65
Age 20‐49	132	26.3	25.56	53	27.4	1.83
Age > 50	235	47	40.83	65	33.7	2.23
Fractures of the hip and the upper leg	248		17.94	111		1.56
Age > 19	13	5.2	4.48	9	8.1	0.68
Age 20‐49	36	14.5	6.97	2	1.8	0.07
Age > 50	199	80.2	34.58	100	90.1	3.43
Fractures of the lower leg	323		23.36	131		1.84
Age > 19	53	16.4	18.28	19	14.5	1.43
Age 20‐49	130	40.3	25.17	54	41.2	1.87
Age > 50	140	43.3	24.33	58	42.3	1.99

Table S1A,B break down the PP of fractures by gender. PP of people with at least one fracture was higher in females than in males in the epilepsy cohort, contrary to the reference cohort, where PP was higher in males than females. Fractures of the lower arm were most prevalent both in males and females in the epilepsy and control group; however, whereas PP was highest in childhood and adolescence and decreased with age among male patients, there was an increase among women aged 50 years and older. PP of hip and femur fractures increase after 50 years of age in PWE and the general population, severely in women with epilepsy. 21 patients had pathological fracture due to osteoporosis and five due to exposure to inanimate forces.

### 3.2 Odds ratios and risk ratios of fractures in relation to ASM

Odds ratios (ORs) and risk ratios (RRs) for different types of fractures among PWE who received one or two ASM and those who received three or more ASM are shown in Table 3. PWE who received more than two ASM during the study period had a higher risk of fractures at various anatomical sites compared with those who received two or fewer ASM (exceptions were hip and femur as well as shoulder and upper arm). Taking 3 or more ASM in men was associated with a 4.5 higher risk of jaw fractures and a 2.1 higher risk of skull fractures (Table S2A). The odds of fracture risk were 1.47 times higher among users of EIASM compared to non‐EIASM users. After controlling for age and gender, no significant differences were found type or treatment (Table S3).

**TABLE 3:** Adjusted and unadjusted odds ratios (ORs) with 95% confidence intervals (CIs) and risk, together with risk ratios (RRs) for different types of fractures among PWE taking 1 or 2 ASM, or more than 2 ASM.

Types of fractures	Numbers of fractures	Unadjusted OR and 95% CI	Adjusted OR^a^ and 95% CI	Risk and RR
Any fracture	1507			RR: 1.32
1 or 2 ASM	1312	ref.	ref	0.10
3 or more ASM	195	1.37 (1.17‐1.61)	1.56 (1.33‐1.84)	0.14
Fractures of the scull	195			RR: 1.94
1 or 2 ASM	160	ref.	ref.	0.013
3 or more ASM	35	1.97 (1.36‐2.85)	1.98 (1.36‐2.88)	0.025
Fractures of the jaw	54			RR: 2.81
1 or 2 ASM	41	ref.	ref.	0.003
3 or more ASM	13	2.83 (1.51‐5.30)	2.80 (1.48‐5.28)	0.009
Fractures of the neck, vertebrae and sacrum	195			RR:1.37
1 or 2 ASM	169	ref.	ref.	0.013
3 or more ASM	26	1.37 (0.90‐2.08)	1.85 (1.21‐2.84)	0.018
Fractures of the shoulder and upper arm	219			RR: 0.94
1 or 2 ASM	198	ref.	ref.	0.016
3 or more ASM	21	0.94 (0.59‐1.48)	1.06 (0.67‐1.67)	0.015
Fractures of the lower arm	501			RR: 1.39
1 or 2 ASM	433	ref.	ref.	0.034
3 or more ASM	68	1.41 (1.09‐1.84)	1.47 (1.13‐1.92)	0.048
Fractures of the hip and upper leg	248			RR: 0.99
1 or 2 ASM	223	ref.	ref.	0.018
3 or more ASM	25	0.99 (0.65‐1.51)	1.76 (1.15‐2.72)	0.018
Fractures of the lower leg	323			RR: 1.29
1 or 2 ASM	282	ref.	ref.	0.023
3 or more ASM	41	1.30 (0.93‐1.81)	1.35 (0.96‐1.88)	0.030

^a^ Analyses adjusted for age.

### 3.3 Fracture risk in relation to the combination of epilepsy and comorbidities

Table 4 presents the impact of comorbidities on the likelihood of fractures in PWE and the control group. The risk for fracture was elevated more than 2‐fold in PWE with osteoporosis and PWE with substance abuse. Out of the other comorbidities investigated, significant increase was seen for dementia and alcohol abuse.

**TABLE 4:** Odds ratio (OR) and 95% confidence interval (CI) for fracture in PWE and controls in relation to different comorbidities.

Comorbidity	Number of individuals	Number of fractures	Unadjusted OR and 95% CI	Adjusted OR^a^ and 95% CI
Any comorbidity				
PWE	5455	702	**1.38 (1.24‐1.54)**	**1.24 (1.10‐1.38)**
Controls	854	565	**4.07 (2.72‐6.06)**	**3.54 (2.36‐5.31)**
Osteoporosis				
PWE	418	103	**2.79 (2.22‐3.51)**	**2.38 (1.87‐3.02)**
Controls	40	3	/	/
Stroke				
PWE	1849	253	**1.35 (1.17‐1.56)**	1.05 (0.90‐1.22)
Controls	297	9	**3.95 (2.02‐7.71)**	**3.23 (1.64‐6.34)**
Dementia				
PWE	854	150	**1.82 (1.51‐2.19)**	**1.35 (1.11‐1.64)**
Controls	98	5	**6.77 (2.74‐16.72)**	**5.27 (2.12‐13.10)**
Substance abuse				
PWE	52	12	**2.46 (1.29‐4.70)**	**2.84 (1.48‐5.43)**
Controls	35	1	/	/
Alcohol abuse				
PWE	232	45	**1.99 (1.43‐2.78)**	**1.81 (1.29‐2.53)**
Controls	35	2	/	/
Intellectual disability				
PWE	791	82	0.94 (0.74‐1.19)	1.15 (0.91‐1.47)
Controls	9	0	/	/
Brain tumors				
PWE	749	75	0.90 (0.70‐1.15)	0.80 (0.62‐1.02)
Controls	55	0	/	/
Movement disorders				
PWE	196	29	1.42 (0.96‐2.12)	1.09 (0.73‐1.63)
Controls	21	0	/	/
Depression				
PWE	967	129	**1.28 (1.06‐1.55)**	1.16 (0.96‐1.41)
Controls	102	3	/	/
Multiple sclerosis				
PWE	27	3	/	/
Controls	22	0	/	/

^a^ Adjusted for age and gender. Bold values represent the statistically significant results.

### 3.4 Incidence rates of different fractures among PWE

The incidence rate of any fracture in PWE was 272.84/10 000 person‐years; higher in the older age group and among people who received 3 or more ASM (Table 5).

**TABLE 5:** Incidence rates of fractures among PWE, stratified by gender, age category, antiseizure medications (ASM) and different types of fractures.

	*N*	Person‐years	Rate/10 000 person‐years
Total number of PWE	1507	55 234.17	272.84
Gender			
Female	686	25 514.525	268.86
Male	821	29 719.645	276.24
Age category			
Age > 19	248	11 588.063	214.01
Age 20‐49	473	20 641.862	229.15
Age > 50	786	23 004.244	341.67
Number of ASM			
1 or 2 ASM	1312	49 642	264.30
3 or more ASM	195	5592.17	348.70
Fractures of the scull	195	55 234.17	35.30
Fractures of the jaw	54	55 234.17	9.78
Fractures of the neck, vertebrae and sacrum	195	55 234.17	35.30
Fractures of the shoulder and upper arm	219	55 234.17	39.65
Fractures of the lower arm	501	55 234.17	90.70
Fractures of the hip and upper leg	248	55 234.17	44.90
Fractures of the lower leg	323	55 234.17	52.44

## 4 DISCUSSION

### 4.1 Principal findings

In this nationwide population‐based cohort study, we found evidence for a higher prevalence of bone fractures in PWE compared to the general population. These data were clear for all age groups, genders, and fracture sites. The landscape of most frequent fracture sites for PWE is similar to the distribution in the general population: lower arm; hip and femur; and lower leg. Prevalence of scull and jaw fractures showed the furthermost difference between PWE and the general population; likely due to falls during seizures. The risk of bone fractures was increased when the group that received more than two ASM was compared separately with the group treated with one or two ASM. Comorbidities had a substantial influence on the likelihood of fractures in PWE.

### 4.2 Strengths and limitations of the study

The major strength of this study is the inclusion of data for the entire epilepsy population in N. Macedonia and matched controls from the general population, utilizing a nationwide electronic health system; therefore, research observations may be considered generalizable. Single‐center studies that investigated the frequency of fractures in selected groups of PWE are limited by a relatively small sample size. Sometimes data were obtained through interviews and questionnaires, which might result in biases due to nonresponse and recall problems. Another strength of our study. is in comparison between the risk of fractures in PWE on one or two ASM vs those who were on more than two ASM, while some previous population‐based studies compared evaluated fracture risk irrespective of number of ASM.^5^, ^26^ We confirmed observations from the older population‐based studies from the United Kingdom and the United States that there is a significant association between markers for epilepsy severity (ie, number of ASM used and seizure frequency) and the risk of fractures; additionally, we evaluated the risk for the landscape of distinct fracture sites.^8^, ^27^

A limitation of our study is that we rely on precoded data and certain data may be imprecise or even missing. For example, we were restricted to fractures requiring medical attention, emergency department visits, or hospitalizations. Some milder fractures might have been missed; for example, some vertebral fractures are not clinically diagnosed. A further limitation of this study is that we did not have data on the fracture etiology. Moreover, our control group was matched by age, gender, and place of permanent residence; hence, we could not exclude confounding effects due to differences in family‐level socioeconomic variables, hereditary risks, and comorbidity. Nevertheless, the increased prevalence of fractures in PWE is likely caused by a combination of factors associated with epilepsy (ie, falls during seizures) and comorbidities of epilepsy known to increase the risk of fractures by itself: stroke, dementia, and osteoporosis.^28^, ^29^

### 4.3 Comparison with other studies and implications for practice

Comparisons of prevalence and incidence rates of all and site‐specific fractures in our study with those of previous work are limited because most studies have been done in specialized epilepsy centers or long‐stay care facilities; or use different methodology (eg, self‐administered questionnaire).^4^, ^15^, ^16^, ^30^, ^31^, ^32^, ^33^ Incidence rates of fractures among PWE in our study was somewhat higher than in population‐based studies using the UK General Practice Research Database (GPRD).^18^ Difference might be due to population included as follows: GPRD data might not include individuals in long‐term care facilities, and that population has a considerably higher risk of falls and fractures; on the contrary, in our study, every PWE who required medical care for a fracture was included.

We found that the prevalence and incidence rates of any fracture in PWE increased with age. Compared to other studies, fractures were more frequent in young and middle‐aged males than in females, while a sharp increase in occurrence was observed among women aged 50 years and older.^18^ Recent population‐based retrospective study using an electronic health register from Sweden found that women aged 75 years and older treated with an inducing ASM against epilepsy and BMIs of 25 kg/m^2^ or below had 48 times higher low energy fractures rates compared to men aged 50 years or younger, treated with a noninducing ASM for a condition other than epilepsy and BMIs above 25 kg/m.^34^

Fracture prevalence was consistently elevated across subtypes in PWE compared to controls, with the most significant difference seen for the skull and jaw fractures. Our finding is consistent with those of population‐based study in Canada about the greatest odds ratio for fractures seen for the head.^26^ Aforementioned UK population‐based cohort study reported the highest risk estimate for hip and femur fractures in PWE compared to controls.^18^ Posterior fracture‐dislocations of the shoulders, thoracic and lumbar vertebral compression, fractures of the skull and jaw, and bilateral femoral neck fractures were most frequently reported in a recent systematic review regarding fractures in association with generalized convulsive seizures in adult patients.^3^ However, this review included dislocation in assessment and also specifically focused on generalized tonic‐clonic seizures.

Finally, the landscape of different comorbidities that increased the likelihood of fractures in PWE is similar to recent Swedish population‐based study about injuries in PWE; nevertheless, we additionally analyzed the effect of osteoporosis on fracture risk.^35^

This study adds comprehensive information about fractures in PWE that might help attempt to establish effective prevention. Real‐world data on epilepsy, fracture events, comorbidities and drug prescriptions as registered in EHR together with basic demographic parameters are a valuable source to identify subgroups with the highest risk and help develop health management guidelines. Although improved seizure control appears the most effective way to reduce risks, preventive measures to improve bone health need to be included.

## 5 CONCLUSION

Our study exploits a large, nationwide inception cohort of PWE to demonstrate that epilepsy patients have an increased all and site‐specific prevalence of bone fractures when compared to matched comparators. The presence of comorbidities and the use of more than two ASM is associated with a higher risk of any fracture.

## AUTHOR CONTRIBUTIONS

MB and BB collected and analyzed patient data and drafted the manuscript for intellectual content. AJ was responsible for statistical analyses and revised the manuscript for intellectual content. IK, MF, and GKT analyzed patient data and revised the manuscript for intellectual content. EC conceptualized the study and revised the manuscript for intellectual content.

## CONFLICT OF INTEREST STATEMENT

The authors have no conflict of interest to disclose. We confirm that we have read the Journal's position on issues involved in ethical publication and affirm that this report is consistent with those guidelines.

## Supporting information


Figure S1.

Table S1.

Table S2.

Table S3.
