# Supplementary material for: Fractures in people with epilepsy: A nationwide population‐based cohort study
Source: Epilepsia Open. 2023 Jun 25;8(3):1028–37. doi: 10.1002/epi4.12776 (PMC10472370; doi:10.1002/epi4.12776)
Supplement: Supplementary file 1 — Figure S1. [file EPI4-8-1028-s002.docx]

﻿

All individuals with epilepsy code

in the National e-Health system 2015-2018

(N=16206)

﻿Excluded (N= 2381): didn’t have code for ASM

﻿

Study population:

6 controls for each PWE , matched for sex, age and place

(N= 71 340)

PWE (N=13.825). 4-6 controls for each PWE , matched for sex, age and place

(N= 71 340)

1507 PWE had a total of 1735 fractures

565 controls had a total of 583 fractures

scull (N=195) scull (N=9)

jaw (N=54 jaw (N=3)

neck, vertebrae and sarcum (N=195) neck, vertebrae and sacrum (N=56)

shoulder and upper arm (N=219) shoulder and upper arm (N=80)

lower arm (N=501) lower arm (N=193)

femur and upper leg (N= 248) femur and upper leg (N=111)

lower leg (N=323) lower leg (N= 131)
