# Supplementary material for: Fractures in people with epilepsy: A nationwide population‐based cohort study
Source: Epilepsia Open. 2023 Jun 25;8(3):1028–37. doi: 10.1002/epi4.12776 (PMC10472370; doi:10.1002/epi4.12776)
Supplement: Supplementary file 2 — Table S1. [file EPI4-8-1028-s001.docx]

**Table S 1. ﻿Period prevalence  of fractures according to location and age group, stratified analyses by gender**

**A. Men**

| **People with epilepsy (PWE), (n= 7 435)** | | | | **Controls, (n= 37 522)** | | |
| --- | --- | --- | --- | --- | --- | --- |
| **Types of fractures** | **Number of fractures** | **%** | **Period prevalence per 1000 inhabitants** | **Number of fractures** | **%** | **Period prevalence per 1000 inhabitants** |
| **People with at least one fractures** | **821** | | **100.4** | **317** | | **8.4** |
| age >19 | 157 | 19.1 | 101.3 | 99 | 31.2 | 3.7 |
| age 20-49 | 285 | 34.7 | 103.8 | 104 | 32.8 | 6.8 |
| age >50 | 379 | 46.2 | 120.7 | 114 | 36.0 | 7.5 |
| **Fractures of the skull** | **127** | | **17.1** | **4** | | **0.1** |
| age >19 | 15 | 11.8 | 9.7 | 0 | 0 | / |
| age 20-49 | 63 | 49.6 | 22.9 | 3 | 75.0 | 0.2 |
| age >50 | 49 | 38.6 | 15.6 | 1 | 25.0 | / |
| **Fractures of the jaw** | **33** | | **4.4** | **2** | | **/** |
| age >19 | 3 | 9.1 | 1.9 | 0 | 0 | / |
| age 20-49 | 23 | 69.7 | 8.4 | 2 | 100 | / |
| age >50 | 7 | 21.2 | 2.2 | 0 | 0 | / |
| **Fractures of the neck, vertebrae and sacrum** | **122** | | **16.4** | **37** | | **1.0** |
| age >19 | 3 | 2.5 | 1.9 | 2 | 5.4 | 0.3 |
| age 20-49 | 44 | 36.0 | 16.0 | 13 | 35.1 | 0.9 |
| age >50 | 75 | 61.5 | 23.9 | 22 | 59.5 | 1.5 |
| **Fractures of the shoulder and upper arm** | **126** | | **16.9** | **37** | | **1.0** |
| age >19 | 18 | 14.3 | 11.6 | 18 | 48.7 | 2.5 |
| age 20-49 | 41 | 32.5 | 14.9 | 6 | 16.2 | 0.4 |
| age >50 | 67 | 53.2 | 21.3 | 13 | 35.1 | 0.9 |
| **Fractures of the lower arm** | **247** | | **33.2** | **126** | | **3.4** |
| age >19 | 94 | 38.0 | 60.6 | 63 | 50.0 | 8.7 |
| age 20-49 | 75 | 30.4 | 27.3 | 42 | 33.3 | 2.8 |
| age >50 | 78 | 31.6 | 24.8 | 21 | 16.7 | 1.4 |
| **Fractures of the hip and upper leg** | **126** | | **16.9** | **48** | | **1.3** |
| age >19 | 8 | 6.4 | 5.1 | 5 | 10.4 | 0.7 |
| age 20-49 | 25 | 19.8 | 9.1 | 2 | 4.2 | 0.1 |
| age >50 | 93 | 73.8 | 29.6 | 41 | 85.4 | 2.7 |
| **Fractures of the lower leg** | **179** | | **24.1** | **72** | | **1.9** |
| age >19 | 32 | 17.9 | 20.6 | 13 | 18.0 | 1.8 |
| age 20-49 | 74 | 41.3 | 26.9 | 38 | 52.8 | 2.5 |
| age >50 | 73 | 70.8 | 54.5 | 21 | 29.2 | 1.4 |

**B. Women**

| **People with epilepsy (PWE), (n= 6 383)** | | | | **Controls, (n= 33 818)** | | |
| --- | --- | --- | --- | --- | --- | --- |
| **Types of fractures** | **Number of fractures** | **%** | **Period prevalence per 1000 inhabitants** | **Number of fractures** | **%** | **Period prevalence per 1000 inhabitants** |
| **People with at least one fractures** | **686** | | **107.5** | **248** | | **7.3** |
| age >19 | 91 | 13.3 | 67.4 | 34 | 13.7 | 5.6 |
| age 20-49 | 188 | 27.4 | 77.7 | 41 | 16.5 | 2.9 |
| age >50 | 407 | 59.3 | 155.6 | 173 | 69.8 | 12.2 |
| **Fractures of the skull** | **68** | | **10.6** | **5** | | **0.1** |
| age >19 | 11 | 16.2 | 8.1 | 2 | 40.0 | / |
| age 20-49 | 32 | 47.0 | 13.2 | 2 | 40.0 | / |
| age >50 | 25 | 38.8 | 9.5 | 1 | 20.0 | / |
| **Fractures of the jaw** | **21** | |  | **1** | | **/** |
| age >19 | 4 | 19.0 | 2.9 | 0 | / | / |
| age 20-49 | 6 | 28.6 | 2.5 | 0 | / | / |
| age >50 | 11 | 52.4 | 4.2 | 1 | / | / |
| **Fractures of the neck, vertebrae and sacrum** | **73** | | **11.4** | **19** | | **0.6** |
| age >19 | 3 | 4.1 | 2.2 | 2 | 10.6 | 0.3 |
| age 20-49 | 23 | 31.5 | 9.5 | 10 | 52.6 | 0.7 |
| age >50 | 47 | 64.4 | 17.9 | 7 | 36.8 | 0.5 |
| **Fractures of the shoulder and upper arm** | **93** | | **14.5** | **43** | | **1.3** |
| age >19 | 18 | 19.4 | 13.3 | 8 | 18.6 | 1.3 |
| age 20-49 | 20 | 21.5 | 8.3 | 4 | 9.3 | 0.3 |
| age >50 | 55 | 59.1 | 21.0 | 31 | 72.1 | 2.2 |
| **Fractures of the lower arm** | **254** | | **39.7** | **67** | | **2.0** |
| age >19 | 40 | 15.8 | 29.6 | 12 | 17.9 | 2.0 |
| age 20-49 | 57 | 22.4 | 23.5 | 11 | 16.4 | 0.8 |
| age >50 | 157 | 61.8 | 60.0 | 44 | 65.7 | 3.1 |
| **Fractures of the hip and upper leg** | **122** | | **19.0** | **63** | | **1.8** |
| age >19 | 5 | 4.1 | 3.7 | 4 | 6.3 | 0.7 |
| age 20-49 | 11 | 9.0 | 4.5 | 0 | / | / |
| age >50 | 106 | 86.9 | 40.5 | 59 | 93.7 | 4.2 |
| **Fractures of the lower leg** | **144** | | **22.5** | **59** | | **1.7** |
| age >19 | 21 | 14.6 | 15.5 | 6 | 10.2 | 0.9 |
| age 20-49 | 56 | 38.9 | 23.1 | 16 | 27.1 | 1.2 |
| age >50 | 67 | 46.5 | 25.6 | 37 | 62.7 | 2.6 |
