# Supplementary material for: Fractures in people with epilepsy: A nationwide population‐based cohort study
Source: Epilepsia Open. 2023 Jun 25;8(3):1028–37. doi: 10.1002/epi4.12776 (PMC10472370; doi:10.1002/epi4.12776)
Supplement: Supplementary file 3 — Table S2. [file EPI4-8-1028-s004.docx]

**Table S 2. Adjusted and unadjusted odds ratios (ORs) with 95% confidence intervals (CIs) and risk, together with risk ratios (RRs) for different types of fractures among PWE taking 1 or 2 antiseizure medications (ASM), or more than 2 ASM. Analyses adjusted for age. Stratified analysis by gender**

1. **Men**

| **Types of fractures** | **Numbers of fractures** | **Unadjusted OR and 95% CI** | **Adjusted OR* and 95% CI** | **Risk and RR** |
| --- | --- | --- | --- | --- |
| **Any fracture** | **821** |  |  | **RR: 1.47** |
| 1 or 2 ASM | 705 | ref. | ref | 0.10 |
| 3 or more ASM | 116 | 1.56 (1.26-1.93) | 1.63 (1.32-2.02) | 0.16 |
| **Fractures of the scull** | **127** |  |  | **RR: 2.19** |
| 1 or 2 ASM | 102 | ref. | ref. | 0.015 |
| 3 or more ASM | 25 | 2.23 (1.43-3.48) | 2.28 (1.46-3.58) | 0.033 |
| **Fractures of the jaw** | **33** |  |  | **RR: 4.47** |
| 1 or 2 ASM | 22 | ref. | ref. | 0.003 |
| 3 or more ASM | 11 | 4.53 (2.19-9.37) | 4.34 (2.08-9.10) | 0.015 |
| **Fractures of the neck, vertebrae and sacrum** | **122** |  |  | **RR:1.25** |
| 1 or 2 ASM | 107 | ref. | ref. | 0.016 |
| 3 or more ASM | 15 | 1.26 (0.73-2.17) | 1.58 (0.91-2.75) | 0.02 |
| **Fractures of the shoulder and upper arm** | **126** |  |  | **RR: 0.94** |
| 1 or 2 ASM | 114 | ref. | ref. | 0.017 |
| 3 or more ASM | 12 | 0.94 (0.51-1.71) | 1.03 (0.56-1.89) | 0.016 |
| **Fractures of the lower arm** | **247** |  |  | **RR: 1.48** |
| 1 or 2 ASM | 212 | ref. | ref. | 0.032 |
| 3 or more ASM | 35 | 1.50 (1.04-2.16) | 1.34 (0.95-1.98) | 0.047 |
| **Fractures of the femur and upper leg** | **126** |  |  | **RR: 1.39** |
| 1 or 2 ASM | 109 | ref. | ref. | 0.016 |
| 3 or more ASM | 17 | 1.40 (0.84-2.35) | 2.06 (1.21-3.50) | 0.022 |
| **Fractures of the lower leg** | **179** |  |  | **RR: 1.66** |
| 1 or 2 ASM | 151 | ref. | ref. | 0.022 |
| 3 or more ASM | 28 | 1.68 (1.12-2.54) | 1.70 (1.13-2.58) | 0.037 |

**B. Women**

| **Types of fractures** | **Numbers of fractures** | **Unadjusted OR and 95% CI** | **Adjusted OR* and 95% CI** | **Risk and RR** |
| --- | --- | --- | --- | --- |
| **Any fracture** | **686** |  |  | **RR: 1.14** |
| 1 or 2 ASM | 607 | ref. | ref | 0.10 |
| 3 or more ASM | 79 | 1.16 (0.90-1.49) | 1.52 (1.17-1.96) | 0.12 |
| **Fractures of the scull** | **68** |  |  | **RR: 1.51** |
| 1 or 2 ASM | 58 | ref. | ref. | 0.010 |
| 3 or more ASM | 10 | 1.52 (0.77-2.99) | 1.47 (0.74-2.91) | 0.015 |
| **Fractures of the jaw** | **21** |  |  | **RR: 0.92** |
| 1 or 2 ASM | 19 | ref. | ref. | 0.003 |
| 3 or more ASM | 2 | 0.92 (0.21-3.98) | 0.96 (0.21-4.17) | 0.003 |
| **Fractures of the neck, vertebrae and sacrum** | **73** |  |  | **RR: 1.56** |
| 1 or 2 ASM | 62 | ref. | ref. | 0.011 |
| 3 or more ASM | 11 | 1.56 (0.82-2.99) | 2.47 (1.26-4.83) | 0.016 |
| **Fractures of the shoulder and upper arm** | **93** |  |  | **RR: 0.94** |
| 1 or 2 ASM | 84 | ref. | ref. | 0.015 |
| 3 or more ASM | 9 | 0.94 (0.47-1.88) | 1.10 (0.55-2.22) | 0.014 |
| **Fractures of the lower arm** | **254** |  |  | **RR: 1.31** |
| 1 or 2 ASM | 221 | ref. | ref. | 0.04 |
| 3 or more ASM | 33 | 1.34 (0.91-1.93) | 1.74 (1.19-2.57) | 0.05 |
| **Fractures of the femur and upper leg** | **122** |  |  | **RR: 0.61** |
| 1 or 2 ASM | 114 | ref. | ref. | 0.019 |
| 3 or more ASM | 8 | 0.61 (0.30-1.25) | 1.42 (0.67-3.00) | 0.012 |
| **Fractures of the lower leg** | **144** |  |  | **RR: 0.87** |
| 1 or 2 ASM | 131 | ref. | ref. | 0.022 |
| 3 or more ASM | 13 | 0.87 (0.49-1.54) | 0.93 (0.52-1.67) | 0.019 |
