# Supplementary material for: Fractures in people with epilepsy: A nationwide population‐based cohort study
Source: Epilepsia Open. 2023 Jun 25;8(3):1028–37. doi: 10.1002/epi4.12776 (PMC10472370; doi:10.1002/epi4.12776)
Supplement: Supplementary file 4 — Table S3. [file EPI4-8-1028-s003.docx]

**Table S 3. Adjusted and unadjusted odds ratios (ORs) with 95% confidence intervals (CIs), risk, and risk ratios (RRs) for different types of ASM.**

| Types of ASM | Number of patients | Number of fractures | Unadjusted OR and 95% CI | Adjusted OR* and 95% CI | Risk | RR | Person years | Rate/10 000 person years |
| --- | --- | --- | --- | --- | --- | --- | --- | --- |
| 1. EIASM monotherapy (carbamazepine, oxcarbazepine, or phenobarbital) | 5928 | 663 | **1.47 (1.26-1.71)** | **1.16 (0.98-1.37)** | 0.11 | 1.37 | 23695.771 | 279.80 |
| non-EIASM monotherapy (levetiracetam, lamotrigine, valproic acid or topiramate) | 3104 | 245 | 0.68 (0.58-0.79) | 0.86 (0.73-1.01) | 0.08 |  | 12407.502 | 197.46 |
| 2. Carbamazepine as monotherapy | 4144 | 472 | **1.31 (1.14-1.50)** | **1.15 (0.99-1.32)** | 0.114 | 1.26 | 16564.655 | 284.94 |
| Other ASM as monotherapy | 4888 | 436 | ref. | ref. | 0.09 |  | 19538.618 | 223.15 |
| 3. Oxcarbazepine as monotherapy | 622 | 51 | 0.79 (0.58-1.06) | 0.80 (0.60-1.08) | 0.08 | 0.8 | 2486.2971 | 205.12 |
| Other ASM as monotherapy | 8410 | 857 | ref. | ref. | 0.10 |  | 33616.975 | 254.93 |
| 4. Phenobarbital as monotherapy | 1163 | 140 | **1.26 (1.05-1.53)** | **1.05 (0.86-1.28)** | 0.12 | 1.23 | 4648.816 | 301.15 |
| Other ASM as monotherapy | 7869 | 768 | ref. | ref. | 0.097 |  | 31454.457 | 244.16 |
| 5. Levetiracetam as monotherapy | 242 | 15 | 0.58 (0.34-0.98) | 0.75 (0.44-1.28) | 0.06 | 0.61 | 967.337 | 155.06 |
| Other ASM as monotherapy | 8790 | 893 | ref. | ref. | 0.10 |  | 35135.935 | 254.15 |
| 6. Lamotrigine as monotherapy | 705 | 60 | 0.82 (0.62-1.08) | 0.87 (0.66-1.14) | 0.08 | 0.83 | 2818.0699 | 212.91 |
| Other ASM as monotherapy | 8327 | 848 | ref. | ref. | 0.10 |  | 33285.203 | 254.77 |
| 7. Valproic acid as monotherapy | 2044 | 163 | 0.73 (0.61-0.87) | 0.95 (0.78-1.14) | 0.08 | 0.75 | 8170.404 | 199.50 |
| Other ASM as monotherapy | 6988 | 745 | ref. | ref. | 0.11 |  | 27932.869 | 266.71 |
| 8. Topiramate as monotherapy | 112 | 7 | 0.59 (0.27-1.28) | 0.67 (0.31-1.44) | 0.06 | 0.62 | 447.693 | 156.35 |
| Other ASM as monotherapy | 8920 | 901 | ref. | ref. | 0.10 |  | 35655.579 | 252.69 |

EIASM - enzyme-inducing antiseizure medications; non-EIASM - non-enzyme inducing antiseizure medications

* Analyses adjusted for age and gender.
